# The *ahpC* c−54t compensatory mutation is not always a valid surrogate for isoniazid resistance in *Mycobacterium tuberculosis*

**DOI:** 10.1128/aac.00265-25

**Published:** 2025-04-22

**Authors:** Viktória Szél, Jody E. Phelan, Sophia B. Georghiou, David L. Dolinger, Taane G. Clark, Stefan Niemann, Lilla K. Lőrinczi, Claudio U. Köser

**Affiliations:** 1 National Reference Laboratory for Mycobacteriology, National Koranyi Institute of Pulmonology 117038 , Budapest, Hungary; 2 Faculty of Infectious and Tropical Diseases, London School of Hygiene & Tropical Medicine 4906, London, United Kingdom; 3 End TB Dx Consulting LLC 12220 , San Diego, California, USA; 4 DLD IVD Consulting LLC, Medway, Massachusetts, USA; 5 Molecular and Experimental Mycobacteriology, Research Center Borstel, Borstel, Germany; 6 German Center for Infection Research, Partner site Hamburg-Lübeck-Borstel-Riems, Borstel, Germany; 7 Department of Genetics, University of Cambridge 98529, Cambridge, United Kingdom; Bill and Melinda Gates Medical Research Institute, Cambridge, Massachusetts, USA

**Keywords:** *Mycobacterium tuberculosis*, isoniazid, genotypic antimicrobial susceptibility testing, *ahpC*, Cepheid Xpert MTB/XDR

## Abstract

Thirteen commercial genotypic antimicrobial susceptibility assays interrogate mutations upstream of *ahpC* to infer isoniazid resistance for *Mycobacterium tuberculosis*. We demonstrate that relying on one of these compensatory mutations (i.e., *ahpC* c−54t)—rather than causative resistance mutations in *katG* that *ahpC* compensates for—can result in systematic false-resistant results for isoniazid with the Cepheid Xpert MTB/XDR and suboptimal treatment. The WHO mutation catalog should be refined to address this scenario.

## INTRODUCTION

With a global prevalence of 11% (95% CI 9–13), isoniazid-resistant, rifampicin-susceptible tuberculosis (Hr-TB) affects approximately three times more new tuberculosis patients than rifampicin-resistant TB (1). Hr-TB treatment requires the inclusion of levofloxacin for 6 months (1).

Cepheid Xpert MTB/XDR (Table 1) is the only WHO-recommended low-complexity automated assay for isoniazid genotypic antimicrobial susceptibility testing (gAST) (2). It analyses two *inhA* promoter regions, responsible for low-level isoniazid and ethionamide/prothionamide cross-resistance, as well as the *katG* codon 315, the most common mechanism of high-level isoniazid resistance (2). Diagnostic sensitivity is further enhanced by targeting the *ahpC* promoter region. Unlike *inhA* and *katG* mutations, mutations that result in the over-expression of *ahpC* act as compensatory mechanisms for fitness defects caused by *katG* mutations rather than directly causing resistance (i.e., they can infer isoniazid resistance when used as surrogates for *katG* resistance mutations outside of the codon 315 region) (3). Notably, the current edition of the WHO mutation catalog does not recognize any *ahpC* mutations as resistance markers of isoniazid because of limitations in its classification algorithm (4). This creates a paradox where a sample may be classified as genotypically resistant due to *ahpC* by the Xpert MTB/XDR but cannot be confirmed by whole genome sequencing (WGS) unless a known *katG* resistance mutation is also present.

**TABLE 1:** Commercial gDST assays that interrogate *ahpC*

Approved for diagnostic use in^*a*^	Assay
European Union^*b*^	Cepheid Xpert MTB/XDR^*c*^ Eudicpia EuDx MDR TB Detection Kit (5) GenoScreen Deeplex Myc-TB^*c*^ ShengTing TBseq (6) Ustar Biotechnologies MultNAT MTC/MDR Assay (7) YD Diagnostics MolecuTech REBA MTB-MDR (8) Zeesan Biotech MeltPro MTB/INH (9) Zeesan Biotech MeltPro MTB/MDR (10) Zeesan Biotech Sanity 2.0 MDR-TB Test Kit (11) Zeesan Biotech Sanity 2.0 MTB/MDR Test Kit (12)
Republic of Korea	Invitros AdvanSure MDR-TB GenoBlot Assay (13)
Russian Federation	BIOCHIP-IMB TB-Biochip-1 (14) BIOCHIP-IMB TB-TEST (15)

^*a*^ Assays may be approved for diagnostic use in additional countries.

^*b*^ CE-IVD.

^*c*^ WHO-recommended (2).

In 2023, the Hungarian National Reference Laboratory for Mycobacteriology received a smear-positive sputum sample that was genotypically susceptible to rifampicin, isoniazid, and ethionamide/prothionamide based on the Bruker/Hain Lifescience GenoType MTBDR*plus* VER 2.0 assay, which does not interrogate *ahpC* (2). The isolate was phenotypically pan-susceptible to all first- and second-line drugs tested, including isoniazid (see Supplementary methods).

A subsequent bronchial aspirate from the same patient was rifampicin-susceptible by Cepheid Xpert MTB/RIF Ultra. Xpert MTB/XDR classified this sample as mono-isoniazid-resistant due to an *ahpC* mutation, even though it was later found to be phenotypically susceptible to isoniazid as well as rifampicin, ethambutol, and streptomycin using the BD MGIT system. Faced with this discordance, isoniazid was removed and, contrary to WHO guidelines, not replaced with levofloxacin, resulting in a regimen of only rifampicin, ethambutol, and pyrazinamide (1). After 2 months of treatment in Hungary, the patient returned to their home country for further treatment.

Subsequent Xpert MTB/XDR testing of the initial culture confirmed genotypic isoniazid mono-resistance. WGS identified the isolate (European Nucleotide Archive accession: ERR13111154) as belonging to lineage 4.1.2.1, a subgroup of the globally widespread Haarlem genotype (16), and carrying a c−54t mutation upstream of the *ahpC* start codon (Table S1). This mutation, located 12 nucleotides upstream of the transcriptional start site, falls within the *ahpC* region targeted by Xpert MTB/XDR, which correctly reported the sample as mutated. Because of the aforementioned limitation of the WHO mutation catalog, this mutation is classified as having uncertain significance, which is also the interpretation provided by the WHO-recommended GenoScreen Deeplex Myc-TB targeted next-generation sequencing gAST assay (Table 1; Alice Ferré, personal communication) (4). Nevertheless, strong experimental evidence from multiple laboratories indicates that this mutation causes *ahpC* overexpression and should, in principle, be a viable surrogate for inferring isoniazid resistance (17–20).

Analysis of ERR13111154 alongside 11,953 other lineage 4.1.2.1 genomes revealed 11 genomes (0.09%) with c−54t as the sole *ahpC* promoter mutation (Table S1). Of these, four also had a *katG* Ser315Thr mutation, and six carried other resistance *katG* mutations (Table S1) (4). Only ERR13111154 lacked mutations in *katG* or its upstream region, resulting in a positive predictive value of *ahpC* c−54t for genotypic isoniazid resistance of 91% (95% CI 59–100) in lineage 4.1.2.1. This is consistent with the positive predictive value of 93% (95% CI 82–100) for phenotypic isoniazid resistance reported by WHO based on a more comprehensive analysis across lineages, suggesting that *ahpC* c−54t is usually a good surrogate for isoniazid resistance (4, 21).

Two hypotheses were explored to explain the discordance with ERR13111154. First, loss-of-function mutations in *ahpC* could theoretically negate the effect of the promoter mutation, similar to how WHO has recognized that *eis* promoter mutations cannot confer amikacin resistance if *eis* is inactive (4, 22). However, no evidence of such epistasis was found (Fig. S1; Table S1). Second, we considered whether an ancestor of ERR13111154 had been isoniazid-resistant due to a *katG* mutation before acquiring the *ahpC* compensatory mutation and then reverting to a susceptible phenotype because of the associated fitness cost (i.e., reverting to wild-type *katG* sequence but retaining the *ahpC* mutation) (22). Phylogenetic analysis found no evidence to support this scenario (Fig. S1; Table S1).

In summary, reliance on compensatory mechanisms such as *ahpC* mutations—rather than causative resistance mutations—can in rare cases lead to systematic false-resistant results and suboptimal treatment decisions. This is relevant for at least 11 other commercial gAST assays that are approved for diagnostic use and interrogate *ahpC* (Table 1) as well as routine WGS diagnostic services globally. These findings highlight the need for revised, evidence-based guidance on *ahpC* in the next edition of the WHO mutation catalog and on how to resolve discordant AST results more generally (2, 23).

## SUPPLEMENTAL MATERIAL

Supplemental material aac.00265-25-s0001.docx Supplemental methods; Fig. S1.

Table S1 aac.00265-25-s0002.xlsx *ahpC*, *katG*, and *inhA* mutations in 11,954 lineage 4.1.2.1 genomes.

ASM does not own the copyrights to Supplemental Material that may be linked to, or accessed through, an article. The authors have granted ASM a non-exclusive, world-wide license to publish the Supplemental Material files. Please contact the corresponding author directly for reuse.
