# Supplementary material for: The ahpC c−54t compensatory mutation is not always a valid surrogate for isoniazid resistance in Mycobacterium tuberculosis
Source: Antimicrob Agents Chemother. 2025 Apr 22;69(6):e00265-25. doi: 10.1128/aac.00265-25 (PMC12135522; doi:10.1128/aac.00265-25)
Supplement: Supplemental material — Supplemental methods; Fig. S1. [file aac.00265-25-s0001.docx]

**Supplementary methods**

*AST*

The culture of the first isolate was found to be pan-susceptible to all first-line drugs (i.e. rifampicin, isoniazid, ethambutol) and second-line drugs tested (i.e. moxifloxacin, levofloxacin, bedaquiline, clofazimine, amikacin and streptomycin) by MGIT using the current WHO critical concentrations (1).

Using the Cepheid Xpert MTB/XDR, the subsequent bronchial aspirate was found to be susceptible to ethionoamide/prothionamide, fluoroquinolones, amikacin, kanamycin and capreomycin but resistant to isoniazid because of *ahpC*. As part of this study, the same result was subsequently obtained performing Cepheid Xpert MTB/XDR on the culture of the first isolate, from which WGS was also carried out (see below).

*WGS*

The culture of the first isolate was sequenced and deposited at the European Nucleotide Archive (ENA) as ERR13111154. It was processed using TB Profiler (v6.4.0) using default parameters to characterise all mutations in drug resistance genes and infer its lineage (i.e. L4.1.2.1) (2). No borderline *rpoB* resistance mutations were identified that might have been missed by MGIT (3, 4).

The SRA accessions of all genomes belonging to L4.1.2.1 were extracted from the TB-Profiler webserver (tbdr.lshtm.ac.uk/sra). Raw data for these accessions were downloaded from the ENA and processed using TB Profiler with default parameters. The 11,954 genomes which were assigned the same lineage L4.1.2.1 were selected for further study. The following genomic regions were interrogated using H37Rv (AL123456.3) as the reference: *ahpC* upstream region (2726101–2726192), *ahpC* coding region (2726193–2726780), *katG* upstream region (2156112–2156642; includes *furA* coding region that was analysed on the nucleotide level), *katG* coding region (2153889–2156111); *inhA* upstream region (1673390-1674201), and *inhA* coding region (1674202–1675011; including the *fabG1* coding region that was analysed on the nucleotide level). Mutations were classified according to TB-Profiler (v6.4.0) using the default profile function, followed by a custom script to extract mutations and their allele frequency (included in parentheses in Table S1).

To identify closely related samples to ERR13111154, a whole genome phylogeny was constructed. In brief, gatk HaplotypeCaller (v4.1.4.1; -ERC GVCF) was used to call variants across the genome for each sample (5). A multi-sample VCF file was created using gatk GenomicsDBImport and gatk GenotypeGVCFs. Custom python scripts (https://github.com/LSHTMPathogenSeqLab/fastq2matrix) were used to convert the VCF into a concatenated SNP alignment in FASTA format. Finally, IQ-Tree (v 2.2.0.3, -m GTR+G+ASC) was used to reconstruct a phylogenetic tree (6). The phylogenetic tree was manually inspected using iTOL and a clade which encompassed ERR13111154 along with 364 other samples (bootstrap = 100) was selected to create the final visualisation of the tree (Figure S1) (7).

**Figure S1**

Phylogenetic tree of 365 lineage L4.1.2.1 genomes, including the Hungarian ERR13111154 strain, which is highlighted in red (a full list of the genomes analysed can be found in Table S1). Group 1/2 isoniazid resistance mutations from the latest version of the WHO mutation catalogue are included (8). Two of the 11 L4.1.2.1 strains that have c−54t as the only *ahpC* upstream mutation are highlighted. In the case of ERR11081547, it occurred with an isoniazid resistance mutation in *katG*, which was also true for the nine strains not shown in this tree (Table S1). By contrast, the Hungarian ERR13111154 strain, had wild-type coding and promoter *katG* regions (i.e. not even a synonymous mutation that might represent a molecular scar due to reversion from a resistant to a susceptible phenotype was present). In addition, ERR11043125, the most closely related strain, was also wild-type for *ahpC* and *katG*. Finally, epistasis could be excluded as a confounder as the *ahpC* coding region of the Hungarian ERR13111154 strain was wild-type (Table S1).

**References**

1. World Health Organization. 2024. WHO operational handbook on tuberculosis: module 3: diagnosis: rapid diagnostics for tuberculosis detection, 3rd ed. <https://iris.who.int/handle/10665/376155>. Retrieved 1 September 2024.

2. Phelan JE, O’Sullivan DM, Machado D, Ramos J, Oppong YEA, Campino S, O’Grady J, McNerney R, Hibberd ML, Viveiros M, Huggett JF, Clark TG. 2019. Integrating informatics tools and portable sequencing technology for rapid detection of resistance to anti-tuberculous drugs. Genome Med 11:41.

3. World Health Organization. 2021. Technical report on critical concentrations for drug susceptibility testing of isoniazid and the rifamycins (rifampicin, rifabutin and rifapentine). <https://iris.who.int/handle/10665/339275>. Retrieved 13 August 2021.

4. Köser CU, Georghiou SB, Schön T, Salfinger M. 2021. On the consequences of poorly defined breakpoints for rifampin susceptibility testing of *Mycobacterium tuberculosis* complex. J Clin Microbiol 59:e02328-20.

5. Van der Auwera GA, O’Connor BD. 2020. Genomics in the Cloud: Using Docker, GATK, and WDL in Terra. 1st Edition. O’Reilly Media.

6. Minh BQ, Schmidt HA, Chernomor O, Schrempf D, Woodhams MD, Haeseler A von, Lanfear R. 2020. IQ-TREE 2: new models and efficient methods for phylogenetic inference in the genomic era. Mol Biol Evol 37:1530–1534.

7. Letunic I, Bork P. 2024. Interactive Tree of Life (iTOL) v6: recent updates to the phylogenetic tree display and annotation tool. Nucleic Acids Res 52:W78–W82.

8. World Health Organization. 2023. Catalogue of mutations in *Mycobacterium tuberculosis* complex and their association with drug resistance, 2nd ed. <https://iris.who.int/handle/10665/374061>. Retrieved 24 February 2024.
